# Robotic adenomectomy using a laparoscopic dissector

**DOI:** 10.1590/S1677-5538.IBJU.2017.0609

**Published:** 2018

**Authors:** Lessandro Curcio Gonçalves, Felipe Monnerat Lott, Rafael Rosa

**Affiliations:** 1 Serviço de Urologia, Hospital Federal de Ipanema, Rio de Janeiro, RJ, Brasil; 2 Departamento de Urologia, Instituto Nacional de Câncer (INCA), Rio de Janeiro, RJ, Brasil

## Abstract

**Introduction:**

Only few reports are known about the use of robotic surgery for prostate benign enlargement. The robotic surgery can be improved by laparoscopic tricks. We show a video of robotic adenomectomy where a laparoscopic dissector is used to help create the plan between prostatic capsule and adenoma.

**Materials and methods:**

A 62 years old male had severe urinary flow outlet obstruction. Medical therapy was not effective. Ultrasound detected a 92gr enlarged prostate with a large middle lobe. Robotic assisted adenomectomy was scheduled. The procedure followed this sequence: opening of Retzius space, superficial suture of the Dorsal vein complex, horizontal cistotomy. The plan was created with electrocautery and blunt dissection with the laparoscopic dissector. Haemostatic sutures were placed between prostate fossa and the posterior bladder neck and closure of the cistotomy.

**Results:**

Whole operation time was 160 minutes, with a blood loss of 80cc. There was no perioperative or post-operative complication. Catheter was removed after 4 days. Post-operatory uroflowmetry shows a peak flow of 30ml/sec. Pathological examination is negative for tumor. After 60 days IPSS was 8.

**Conclusion:**

Robotic prostate adenomectomy using the laparoscopic dissector is a safe and effective minimally invasive treatment for benign prostatic enlargement. It is a novel technique to find and dissect the plane between prostatic adenoma and capsule. This could be one more use of laparoscopic technology to improve surgical outcomes in robotic field.

## ARTICLE INFO

**Available at: http://www.intbrazjurol.com.br/video-section/20170609_Goncalves_et_al**

**Int Braz J Urol. 2018; 44 (Video #17): 1051-1051**

